# Intraocular lens dislocation: manifestation, ocular and systemic risk factors

**DOI:** 10.1007/s10792-022-02529-6

**Published:** 2022-09-23

**Authors:** Jana Catharina Riedl, Severin Rings, Alexander K Schuster, Urs Vossmerbaeumer

**Affiliations:** Department of Ophthalmology, University Medical Center, Johannes Gutenberg-University Mainz, University Hospital Mainz, Langenbeckstraße 1, 55131 Mainz, Germany

**Keywords:** Intraocular lens dislocation, Intraocular lens, Pars plana vitrectomy, Secondary lens implantation, OCT

## Abstract

**Purpose:**

The aim of this study was to evaluate ocular and systemic risk factors for posterior chamber intraocular lens dislocation, as well as forms of manifestation.

**Methods:**

A retrospective case–control study were all patients presented in the period 2012–2016 having intraocular lens dislocation and being treated with implantation of an iris-fixated intraocular lens was conducted at the University Hospital Mainz. As controls, pseudophakic patients presenting for other reasons were included.

**Results:**

150 eyes of 150 patients (mean age 72.7 ± 12.4 years, range 24–93 years) with IOL dislocation and 150 eyes of 103 controls were included in this study. The average time between primary implantation and IOL luxation was 86 months (iQR: 39.25–127 months) for all dislocations. Previous pars plana vitrectomy (PPV) (crudeOR = 2.14 (95% CI 1.23, 3.72), *p* = 0.011) and PEX (crudeOR = 11.6 (4.79, 28.12), *p* < 0.001) was linked with a higher risk of IOL luxation. Luxation occurs also earlier in patients with previous PPV and PEX than in eyes with neither PEX nor previous PPV (82.2 vs. 127 months). Rhegmatogenous retinal detachment was the major pathology that required a previous PPV for eyes with an IOL dislocation (57%). The average time between PPV and IOL dislocation was 74.67 months (range 0–186 months).

**Conclusion:**

Patients with a coexistence of both: PEX and a previous PPV had an elevated risk of IOL dislocation, and also had a shorter time interval between primary IOL implantation and IOL dislocation followed by eyes with PEX only and eyes with only a previous PPV.

## Introduction

The dislocation of intraocular lenses (IOL’s) is a rare but serious complication after cataract surgery [1]. It typically does not occur in the early postoperative phase, but at a later stage, which is known as “late-in-the-bag-dislocation.” Late-in-the-bag-dislocation is defined as a spontaneous dislocation more than three months (usually occurring several years later) after cataract surgery and it is due to a slowly decrease in the stability of the zonules [2–4]. Some potential risk factors have already been described in the literature. Pseudoexfoliation (PEX) is mentioned as the most common cause, alongside high myopia, previous vitreoretinal surgery, trauma, aging, connective tissue disorder, retinitis pigmentosa, uveitis, diabetes mellitus and retinitis pigmentosa [1–6]. Previous pars plana Vitrectomy (PPV) is often described [1, 2], but the typical latency period between PPV and the IOL luxation is not known and should be defined more precisely. The reported incidence of dislocation ranges between 0.2 and 2% [3]. In addition, men are more likely to be affected than women, for which no plausible reason has yet been found [3, 7–9]. The number of IOL dislocation has increased noticeably over the past few years, although it is not yet clear whether this is due to an increased rate of incidence of IOL dislocation or whether it is due to the increase in the number of pseudophakic subjects in the population [1, 10]. The therapy always includes a surgical revision, whereby the IOL can sometimes be repositioned, but often needs to be completely replaced. In such a case, an iris-fixated lens can serve as a replacement.

The aim of this retrospective case–control study is to evaluate ocular and systemic risk factor for IOL dislocation. Furthermore, the typical latency period until dislocation, especially after vitrectomy, was evaluated.

## Materials and methods

This retrospective case–control study was performed at the Department of Ophthalmology, University Medical Center of the Johannes Gutenberg-University Mainz. According to local law (“Landeskrankenhausgesetz” §36, §37), no ethical approval was required for this retrospective analysis. On the basis of operation reports, patients been treated for IOL dislocation and having had received a secondary lens implantation between 2012 and 2016 were identified.

The inclusion criteria were: secondary lens implantation after (sub-) luxation of the primary intraocularlens (IOL) < 180 months after the primary surgery, complete medical record. The exclusion criteria were: incomplete medical report, primary implantation of an iris-fixated intraocularlens, aphakia with iris-fixated IOL implantation, secondary lens implantation after explanation of an opacificated IOL, re-enclavation of subluxated iris-fixated IOL, and secondary lens implantation after (sub-)luxation of the primary IOL > 180 months after the primary cataract surgery. As control group, a pseudophakia (after cataract surgery, with intraocular lens) patient cohort from the medical retinae center of the University Hospital Mainz were included with an age- and “timepoint of cataract surgery” matching with a 1:1 ratio (control group:study group).

The following data were collected: date of the primary cataract surgery, type of luxation (in the bag/ out of the bag), axial length, history of eye disease, previous pars plana vitrectomy, pseudoexfoliation, systemic disease (diabetes mellitus, coronary artery diseases, arterial hypertension, obstructive pulmonary disease).

### Statistical methods

For descriptive analyses, mean and standard deviation were calculated for approximately normal distributed data, otherwise median and interquartile range. For categorical variables, absolute and relative frequencies were computed. Logistic regression analyses were carried out to analyze potential risk factors for IOL dislocation, including age, gender, previous PPV, PEX, diabetes mellitus, arterial hypertension, obstructive pulmonary disease. A *p*-value < 0.001 was considered as strong association, *p* < 0.05 as likely association, *p* > 0.05 but < 0.6 as inconclusive and *p* ≥ 0.6 as probably not associated. A Kaplan–Meyer curve was used to analyze the time point of the intraocular lens dislocation after primary implantation regarding PEX and previous PPV.

Statistical analysis was carried out using SPSS 23.5 software (IBM Corp., IBM SPSS Statistics for Macintosh, version 24.0, Armonk NY) and R (R Core Team 2020). R: a language and environment for statistical computing. R Foundation for Statistical Computing, Vienna, Austria. URL http://www.R-project.org/).

## Results

### Patient collective

In this study, we analyzed 299 eyes of 299 consecutive patients who had surgery under general anesthesia with implantation of a secondary intraocular lens between 2012 and 2016. In 7 eyes, incomplete medical data existed. Furthermore, 142 eyes were excluded because of other exclusion criteria (36 eyes with primary iris-fixated IOL implantation, 8 eyes with IOL opacification, 47 eyes with aphakia and iris-fixated IOL implantation, 12 eyes with re-enclavation of the iris-fixated IOL). Thus, 150 eyes of 150 patients met the inclusion criteria.

**Fig. 1:**
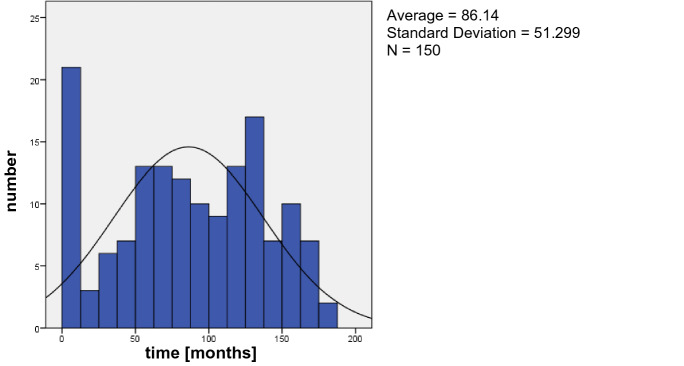
Time (months) between primary Cataract Surgery and IOL dislocation

**Fig. 2:**
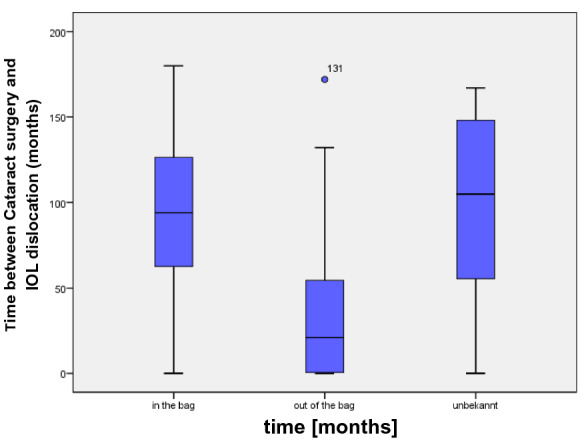
Time between primary Cataract Surgery and IOL dislocation according to the type of luxation

In the study group, 97 patients (64.67%) were male and 53 patients (35.3%) were female with an average age of 72.7 ± 12.4 years (range 24–93 years). The average age of the control group was 73.67 ± 12.8 years (range: 22–95 years) with 63 (42%) male and 87 (58%) female patients, Table 1. The average time between primary cataract surgery and IOL dislocation was 86.1 months (= 7.18 years) with a range of 0–180 months, Fig. 1. Based on the available surgical reports, the implantation of the lens during the primary cataract surgery could be determined in 131 eyes. 108 IOL’s were implanted in the bag; 23 IOL’s were implanted in the ciliary sulcus. The average time between primary implantation and luxation was 94 months (7.84 years) for in-the-bag luxation and 37.13 months (3.1 years) for out-of-the-bag luxation, Fig. 2. Seven patients (4.7%) had a previous blunt ocular trauma, while there was just one blunt ocular trauma (0.7%) in the control group. Some specific ocular characteristics and systemic diseases are shown in Table 2.

**Table 1:** Characteristics of patients with IOL luxation and the control group

	Patients with IOL luxation	Control group
Age (years)	72.9 ± 12.4	74.4 ± 12.8
	(24–93)	(22–95)
Gender (f/m)	53/97	87/63
Capsular tension ring (*n*)	18	0
PEX	49	0
PPV	46	6

**Table 2:** Logistic regression analysis for IOL dislocation with respect to pseudoexfoliation (PEX), previous pars plana vitrectomy, arterial hypertension, diabetes mellitus and obstructive pulmonary disease

	Study group (%)	Control group (%)	Crude OR (95% CI)	*p*-value	Multivariable OR (95% CI)	*p*-value
PEX	32.7	4.0	11.61 (4.3, 28.1)	< 0.001*	17.43 (6.8, 44.9)	< 0.001*
Previous pars plana vitrectomy	30.7	16.7	2.14 (1.2, 3.7)	0.011*	2.22 (1.2, 4.1)	0.011*
Arterial hypertension	60.7	66.0	0.8 (0.5, 1.3)	0.76	0.92 (0.5, 1.6)	0.76
Diabetes mellitus	24.0	31.3	0.69 (0.4, 1.2)	0.059	0.56 (0.3, 1.0)	0.056
Obstructive pulmonary disease	12.0	10.7	1.14 (0.6, 2.3)	0.67	1.2 (0.5, 2.8)	0.67

The multivariable analysis was also adjusted for age and gender

^*^Significant; + 146 eyes of the study group and 99 eyes of the control group were included

**Fig. 3:**
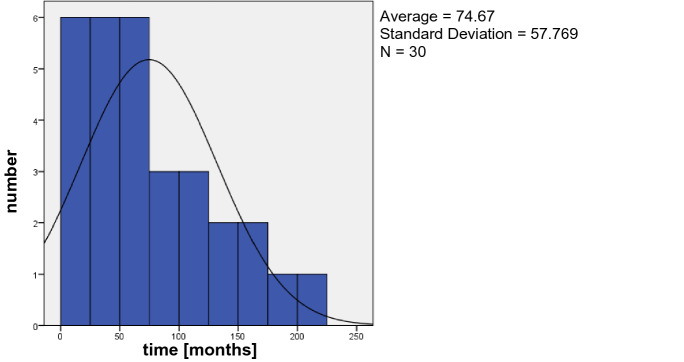
Time (months) between pars plana Vitrectomy and IOL dislocation (study group)

**Fig. 4:**
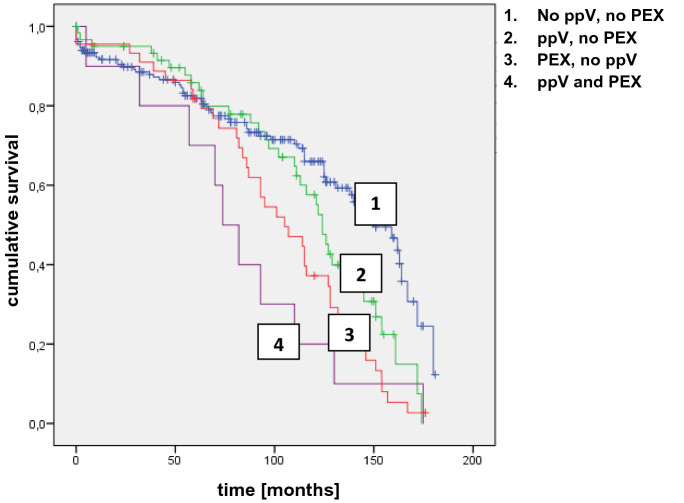
Kaplan–Meier curve. Patients with both -previous pars plana vitrectomy and PEX- are most likely to develop an IOL luxation at an early stage (group 4). In contrast, patients without this both risk factors are less likely for an IOL luxation (group 1)

PEX and previous PPV show a statistical significance between the study group and control group. For 30 eyes in the study group (23 eyes of the control group) an evaluation of the time between pars plana vitrectomy and IOL dislocation was possible and is shown in Fig. 3 for the study group. The average time between PPV and IOL dislocation was 74.67 months. The features PEX and pars plana vitrectomy were further analyzed and illustrated using a Kaplan–Meyer curve, Fig. 4. An IOL dislocation occurs not only more frequently but also earlier in patients with a coincidence of a previous pars plana vitrectomy and PEX than in patients without both features. Compared with patients with neither a previous pars plana vitrectomy nor PEX the difference of the median was 74 months versus 150 months and the mean was 82.2 months (group 4) versus 127 months (group 1). Group 2 (previous PPV, no PEX) showed a mean of 116 months and a median of 124 months; in group 3 (PEX, no PPV) the mean was 101 months and the median 105 months. Furthermore, the logarithmic probability of IOL dislocation was calculated (Table 1). According to this, the crude Odds-ratio for PEX is 11.6 (multivariable OR 17.4) and 2.14 (multivariable OR 2.22) for eyes with a previous pars plana vitrectomy.

In 31 eyes it was possible to evaluate the cause of the previous PPV: 28 eyes had undergone the previous vitrectomy for rhegmatogenous retinal detachment (57%), 3 eyes for membrane peeling (6%) and 18 eyes remained unknown (37%).

According to the medical reports, 18 eyes (12%) had an implantation of a capsular tension ring (CTR) during the cataract surgery. Furthermore, 23 eyes (15.3%) had IOL implantation in the ciliary sulcus during the cataract surgery. The IOL’s with sulcus implantation luxated significantly earlier (*p* = 0.006) than the IOLs with capsular bag implantation (37.1 ± 48.1 months vs. 95.2 ± 48.0 months), while there was no difference between IOL’s with CTR and IOLs without CTR (*p* = 0.69; 86.6 ± 45.4 months vs. 87.5 ± 52.3 months).

## Discussion

This present study evaluates ocular and systemic risk factors for an IOL dislocation after cataract surgery. A coincidence of PEX and previous pars plana vitrectomy increases the risk of an IOL dislocation. In addition, in such cases a dislocation takes place much earlier in contrast to the presence of only one of these risk factors. The average time between IOL implantation and IOL dislocation was 86.1 months. However, lenses with a primary sulcus implantation had a significantly shorter survival time (37.1 months) than IOL’s which were primary implanted in the capsular bag (94 months).

Our study population consisted of 2/3 male patients and 1/3 female patients with an average age of 72.7 years, which is similar to the previous studies of Subasi et al. and Krepste et al. [2, 11]. A reason for the noticeably higher participation of men, as also described in other studies [1–3, 9, 12], has not yet been found. More women than men undergo cataract surgery and have PEX [1, 13], but there may be a difference in a weaker zonula in men with PEX than in women [7].

The overall follow-up time was 180 months (15 years) in this study. Patients with a IOL dislocation after 180 months were excluded, because of a missing control group. As illustrated in Fig. 1, there is a peak of dislocation in the first year and after 130 months (5.4 years). The average time between IOL implantation and dislocation was 86.1 months (7.1 years). The mean time interval from cataract surgery to dislocation varies from 7.1 to 8.5 years in the literature, which is similar to our findings [2, 5, 7, 14]. As presented in Fig. 4, patients with both-previous pars plana vitrectomy and PEX- are more likely to develop an IOL dislocation at an early stage (group 4) followed by PEX only. With a previous PPV the Odd-ratio of an IOL dislocation was 2.14 in our study compared to the control group and remarkable 11.6 in PEX eyes. PEX was the most important risk factor in our study. It was described in 32.7% (49 eyes) of the patients. It is also always the first or second important risk factor in the literature but with a remarkable high variance of 16.4% up to 66.6% in different studies [7, 15]. PEX material accumulations is already known for mechanically weaken the zonular and there is also an increased elastinolysis causing enzymatic zonular friability [16–18]. To minimize the risk of an IOL dislocation in PEX eyes after cataract surgery, it is advisable to be operated by experienced surgeons [16]. PEX in combination with a previous PPV shows the highest risk of IOL dislocation in our study. Aggressive peripheral vitrectomy with scleral depression and manipulation of the vitrectomy ports may damage the zonules [14, 19, 20], which is even more likely in patients with PEX and the resulting zonular friability. Like Koike et al., we also could state that rhegmatogenous retinal detachment was the major pathology that required PPV for eyes with an IOL dislocation followed by PPV for a membrane peeling [20]. While this is a retrospective study, we could not evaluate the reason for the previous pars plana vitrectomy in all patients.

In contrast to Fernandes-Buenaga et al. and Tran THC et al., who described high myopia as the most prevalent risk factors, the axial length had no significant effect in our study [7, 21]. High myopia has also already mentioned in some single case reports as a risk factor, but we could not find any major discrepancies between the study group and control group [6, 21]. The mean axial length in the study group was 24.66 mm (range: 20.82–34.05 mm) and 23.94 mm (range: 12.02–31.48 mm) in our control group (*p*-value 0.059). Of note, there is no statistic significant but as the axial length of the study group corresponds to an average refraction of moderate myopia, it could be a slight note for myopia as a risk factor for in-the-bag dislocation according to the literature.

In the existing literature, ocular trauma is discussed as a risk factor for lens luxation [1, 22, 23]. A preoperative trauma can lead to zonular dehiscence and also capsular contraction [1]. In this study, only a few patients had a preoperative surgical trauma (7 out of 150 patients). In contrast, pseudoexfoliation was described in 49 eyes. This is consistent with the existing literature, in which pseudoexfoliation is described as the most common risk factor for zonular dehiscence [1]. The IOL’s with sulcus implantation luxated significantly earlier than the IOLs with capsular bag implantation, while there was no difference between IOL’s with CTR and IOLs without CTR. This is not in line with the findings of Stuermer 2013, who described significantly earlier luxation in patients with CTR implantation than in IOLS without CTR [24]. In the literature, neither the IOL implantation in the ciliary sulcus nor the implantation of a CTR are described as risk factors for IOL luxation. Tribus et al. evaluated 69 eyes with a CTR and described just one case with luxation in the vitreous body and 5 eyes with a dislocation of the IOL without the need for further surgery [25]. Complications for ciliary sulcus implantations are secondary pigment dispersion, elevated IOP, secondary pigmentary glaucoma, intraocular hemorrhage and iris transillumination defects [26].

There are various options for secondary lens Implantations. Depending on the initial situation, the best possible option should be selected for the patient. IOL implantation in the sulcus with and without optic capture has the lowest complication rate and best visual acuity outcome [27]. Scleral fixation is often used in complex or traumatic cases without adequate capsule support, when other options are not feasible [28, 29]. Iris-claw lenses have gained more and more popularity over the last years with the advantages of small learning curve and low complication rates [30]. They can be placed in front of the iris or retropupillar [30]. Only patients with a secondary retropupillary iris-fixated IOL implantation were included in this study to analyze a homogeneous study group.

This study design also has some limitations. As shown in Table 1, patients with diabetes mellitus have a lower risk for IOL dislocation compared to the control group. This is probably due to our selection of the control group, which are patients from the medical retinae center. Here, patients with macular edema receive intravitreal injections, although of course more diabetes patients are among them. Therefore, this value is not usable. Furthermore, patients having a secondary lens implantation after (sub-)luxation of the primary IOL > 180 months after the primary cataract surgery had to be excluded because of missing controls. Moreover, seven patients were excluded because of a missing or incomplete medical record. Thus, there is the risk of selection bias. In addition, not all patients had the primary cataract operation in our institution. So, we were reliant on external medical report/anamneses of the patients.

## Conclusion

Patients with a coexistence of PEX and previous PPV not only have an increased risk of IOL dislocation, but also have the shortest time interval between primary IOL implantation and IOL dislocation followed by eyes with PEX only and eyes with only a previous PPV. The average time between PPV and IOL dislocation was 74.67 months. Rhegmatogenous retinal detachment was the major pathology that required PPV for eyes with an IOL dislocation (57%). While IOL implantation in the sulcus had a significant impact on the time between primary cataract surgery and IOL luxation, implantation of a CTR showed no difference.
